# Supplementary figures and images for: Unraveling the significance of decorin in endometriosis development through single cell sequencing and experimental approaches
Source: PLoS One. 2026 May 15;21(5):e0349505. doi: 10.1371/journal.pone.0349505 (PMC13178861; doi:10.1371/journal.pone.0349505)

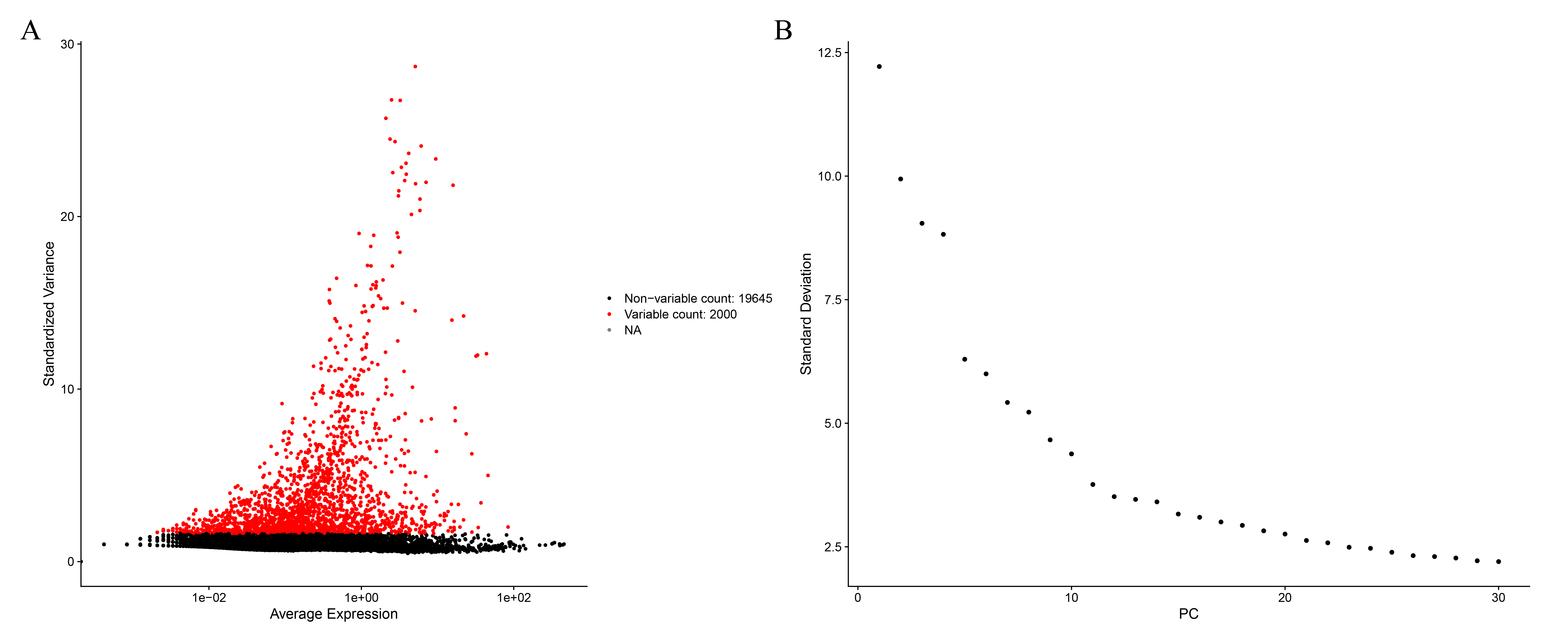

Supplement: S1 Fig — (A) Top 2,000 highly variable genes. (B) Top 30 principle components (PCs). (TIF) [file pone.0349505.s001.tif]

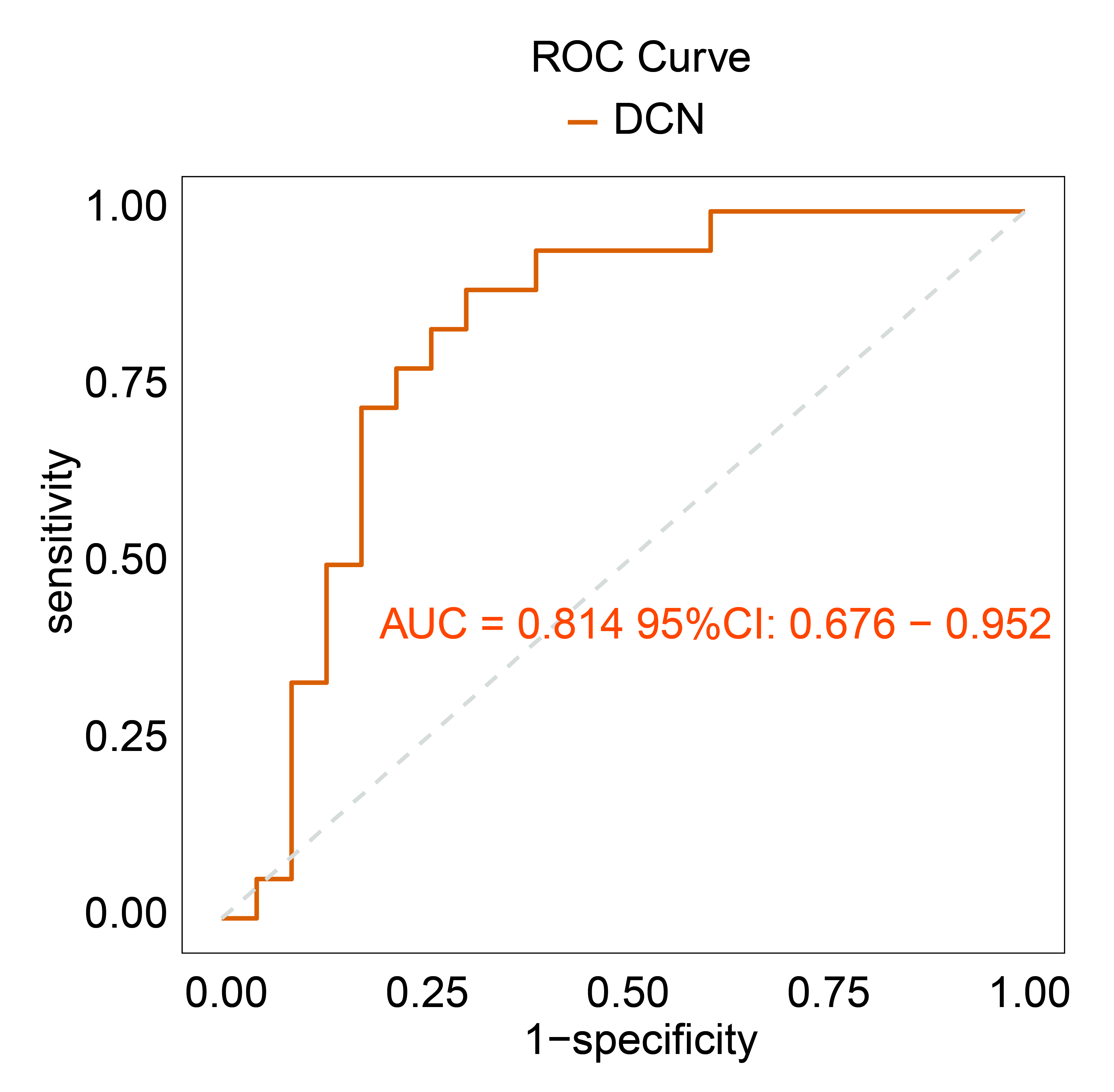

Supplement: S2 Fig — (TIF) [file pone.0349505.s002.tif]
