# Supplementary material for: Unraveling the significance of decorin in endometriosis development through single cell sequencing and experimental approaches
Source: PLoS One. 2026 May 15;21(5):e0349505. doi: 10.1371/journal.pone.0349505 (PMC13178861; doi:10.1371/journal.pone.0349505)
Supplement: S1 Table — (DOCX) [file pone.0349505.s003.docx]

**Supplementary Table 1. The sequences for primers**

| **Primers** | **Sequences** |
| --- | --- |
| DCN | F:GGAATTGAAAATGGGCTTT |
|  | R:GCCATTGTCAACAGCAGAGA |
| GAPDH | F:TCTTGGGCTACACTGAGGAC |
|  | R:CATACCAGGAAATGAGCTTGA |
