# Supplementary material for: Unraveling the significance of decorin in endometriosis development through single cell sequencing and experimental approaches
Source: PLoS One. 2026 May 15;21(5):e0349505. doi: 10.1371/journal.pone.0349505 (PMC13178861; doi:10.1371/journal.pone.0349505)
Supplement: Attachment — Submitted filename: Response to Reviewers.docx [file pone.0349505.s007.docx]

**Point-by-point response to the reviewer comments**

**Dear Reviewers,**

Thank you for your thoughtful suggestions and insights, which have benefited from the manuscript. I am looking forward to working with you to move this manuscript closer to publication in “**PLOS One**”.

The manuscript has been rechecked and the necessary changes have been made in accordance with your suggestions. The responses to all comments have been prepared and attached below. We have tried our best to solve the problems you proposed, and we hope that the revised manuscript is now suitable for publication in the journal “**PLOS One**”. If you have any questions remained about this paper, please feel free to contact us.

Reviewer #1: The manuscript by Chen et al. investigates the role of Decorin (DCN) in endometriosis by integrating single-cell RNA sequencing, bulk transcriptomic deconvolution, and in vitro validation. The study addresses a relevant and timely topic, given the unmet need for reliable biomarkers and mechanistic insights into endometriosis pathogenesis. The combination of scRNA-seq-based stromal cell subtyping with experimental validation represents a notable strength.

Overall, the manuscript is interesting, methodologically solid, and potentially suitable for publication, but several conceptual, methodological, and presentation issues need to be addressed before acceptance.

Major comments

1. Conceptual clarity on the role of DCN (biomarker vs functional driver)

While the study convincingly demonstrates differential expression and diagnostic potential of DCN, the manuscript repeatedly refers to DCN as a “promoter of endometriosis progression”

However:

• The functional experiments are limited to expression validation (RT-qPCR).

• No loss-of-function or gain-of-function assays (e.g., DCN knockdown, overexpression, rescue experiments) are presented.

• No direct mechanistic assays (e.g., migration, invasion, fibrosis markers, TGF-β signaling readouts) are shown.

RE: We sincerely appreciate you for the careful and professional review of our manuscript, as well as the valuable and constructive comments on the description of DCN function. We fully agree with the reviewer’s reasonable concern that although our study convincingly demonstrates the differential expression and promising diagnostic potential of DCN in endometriosis, the manuscript describes DCN as a “promoter of endometriosis progression” without sufficient functional and mechanistic evidence.

The main purpose of the present study was to identify candidate biomarkers associated with endometriosis through comprehensive bioinformatics analysis, and to verify the differential expression of DCN using RT‑qPCR, so as to provide a novel potential diagnostic index and lay a preliminary theoretical foundation for subsequent functional and mechanistic research on DCN in endometriosis. Due to current limitations in research resources, experimental conditions, and research timeline, we have not yet performed loss‑of‑function or gain‑of‑function experiments (such as DCN knockdown, overexpression, and rescue assays), nor direct mechanistic investigations including cell migration, invasion, fibrosis marker detection, and TGF‑β signaling pathway analyses. We fully recognize this limitation of the current study. Therefore, we have revised the relevant descriptive statements in the manuscript to avoid overstating the functional role of DCN as a “promoter of endometriosis progression” without direct experimental evidence **(Pages 4-5, Lines 73-86, Pages 17-18,Pages 13-14, Lines 273-296, Pages 17-18, Lines 357-379)**. Meanwhile, we have supplemented a detailed discussion of this limitation in the Discussion section **(Pages 17-18, Lines 357-379)**.

We hope these revisions adequately address your concern and greatly improve the rigor and accuracy of the manuscript. Thank you again for your insightful and helpful feedback.

2. Stromal subcluster annotation requires stronger validation

The identification of 10 stromal subtypes is a central result.

However:

• Functional annotation is based mostly on enrichment analysis rather than well-established stromal markers. Provide a clear table of top 5–10 marker genes per subtype to support annotations.

• Names such as Stemness-Remodeling Stromal Cells and Metabolic-Endocrine Dual-Function Stromal Cells are introduced without literature precedent. Clarify that these names represent functional states rather than definitive identities, or move them to supplementary material.

• The manuscript does not sufficiently compare stromal subtypes with previously described endometrial stromal populations. Explicitly compare the identified subtypes with published human endometrium or endometriosis scRNA-seq datasets to strengthen biological validity.

RE: We sincerely appreciate your valuable and professional comments on the annotation of stromal cell subclusters, which are crucial for optimizing the rationality and reliability of our cell subtype definition. We have revised the manuscript accordingly and respond to each point as follows.

a. Regarding functional annotation and marker gene presentation

We agree that sufficient marker genes are necessary to support stromal subtype annotation. In the revised manuscript, we have supplemented a clear table showing the representative marker genes for each stromal subtype. Meanwhile, the complete marker gene list for each cluster (obtained by intersecting highly variable genes and functionally enriched genes of each cluster) is provided in the **Supplementary Table 2** for further validation **(Page 11, Lines 212-213)**.

b. Regarding the naming of stromal cell subtypes

In the re‑annotation of stromal cells, the annotation of several major clusters was strictly based on previously published studies: Cluster 4/12 were annotated as proliferative endometrial stromal cells (estromal) (PMID: 39198675); Cluster 1/2/3/8 were annotated as decidualized stromal cells (dstromal) (PMID: 39198675); Cluster 6 was annotated as vascular smooth muscle cells (VSMC) (PMID: 41574608); Cluster 7 was annotated as pericytes (PMID: 34067358).

To further explore the potential biological characteristics of the remaining clusters, we performed functional enrichment analysis based on their highly variable genes and adopted descriptive names according to their enriched functions. These names only represent predicted functional states of the corresponding cell clusters, rather than canonical or definitive cell identities **(Pages 11, Lines 213-227)**.

c. Regarding comparison with published scRNA-seq datasets of human endometrium or endometriosis

We apologize for not explicitly comparing our identified stromal subtypes with previously published human endometrial and endometriosis scRNA-seq datasets in the original manuscript.

In the revised version, we have compared the marker genes and functional enrichment profiles of our stromal subclusters with well-characterized stromal populations reported in previous single-cell studies. Our major annotated clusters, including proliferative endometrial stromal cells (estromal, Clusters4/12) (PMID: 39198675), decidualized stromal cells (dstromal, Clusters1/2/3/8) (PMID: 39198675), vascular smooth muscle cells (VSMC, Cluster6) (PMID: 41574608) and pericytes (Cluster7) (PMID: 34067358), showed high consistency in signature gene expression and biological functions with canonical endometrial stromal subtypes described in the literature. The remaining clusters with custom functional names represent newly identified cell states rather than previously defined cell types **(Page 11, Lines 213-218)**.

We believe the above revisions have fully addressed your concerns and improved the rigor of the manuscript.

3. Inconsistencies in immune terminology

• The manuscript refers to immune microenvironment analysis, but deconvolution focuses mainly on stromal subtypes, not classical immune cells.

• Terms such as “immune cell proportion” are used inconsistently (Figures 4C-D).

Clarify terminology throughout. Use “cellular composition” or “stromal composition” instead of immune cells where appropriate.

Justify the use of CIBERSORTx for stromal deconvolution, as it is typically applied to immune populations.

RE: We sincerely appreciate your careful and constructive comments on the terminology consistency and methodological rationality regarding deconvolution analysis. We have carefully revised the manuscript and addressed each concern in detail as follows.

a. Regarding inconsistency between immune microenvironment description and stroma‑focused deconvolution

We apologize for the inappropriate description of immune microenvironment in the original manuscript. As the deconvolution analysis in this study mainly focused on stromal cell subtypes rather than classical immune cell populations, we have revised the relevant descriptions throughout the manuscript to accurately reflect the analytical content **(Page 12, Lines 237-250)**.

b. Regarding inconsistent use of terms such as “immune cell proportion”

We have carefully checked and unified all inconsistent terms in the text, figures, and figure legends. The misleading term “immune cell proportion” has been replaced with “cellular composition” where appropriate, ensuring clarity and consistency in terminology.

c. Justification for using CIBERSORTx for stromal deconvolution

We fully agree that the rationality of method application should be explicitly clarified. CIBERSORTx is built on support vector regression (SVR) and a linear deconvolution framework, which is not inherently restricted to immune cell populations. In contrast to the original CIBERSORT algorithm that uses a fixed immune cell signature matrix (LM22), CIBERSORTx supports custom signature matrices derived from user‑provided single‑cell RNA‑seq data. In this study, we constructed a stromal cell‑specific signature matrix from our scRNA‑seq data, enabling accurate deconvolution of stromal subtype composition. We have added this justification in the revised Methods and Result section to clarify the validity of applying CIBERSORTx to stromal deconvolution **(Pages 7-8, Lines 137-149, Page 12, Lines 237-250)**.

We believe these revisions have greatly improved the accuracy and rigor of the manuscript.

4. Diagnostic value of DCN: limited clinical applicability

o ROC analyses show good AUC (>0.8), but sample sizes are small, particularly for controls (e.g., GSE135485).

• No independent clinical validation cohort is included.

• Tissue-based expression may limit clinical translation.

Avoid overstatement of DCN as a diagnostic biomarker.

Discuss potential non-invasive applications, e.g., detection in serum, peritoneal fluid, or menstrual effluent.

RE: We sincerely appreciate your professional and rigorous comments on the diagnostic value of DCN. These suggestions help us to present our results more objectively and improve the translational perspective of this study. We have revised the manuscript accordingly and respond as follows.

a. Regarding small sample size in the control group of GSE135485

We acknowledge the relatively small number of control samples in the GSE135485 dataset. To improve the reliability of ROC analysis, we have supplemented ROC curve analysis for DCN using another independent public dataset GSE7307 (**Supplementary Figure 2**). This dataset includes 41 endometrial tissue samples, with a balanced sample size (endometriosis: n = 18, control: n = 23; microarray platform: GPL570). Although GSE135485 contains only four control samples, consistent results across multiple datasets support the reliability of our findings **(Pages 5-6, Lines 89-100, Page 13, Lines 259-261)**.

b. Regarding the lack of an independent clinical validation cohort

We agree with the reviewer that the absence of a prospectively collected independent clinical validation cohort is a notable constraint. In the revised manuscript, we have explicitly listed the lack of an independent clinical validation cohort as one of the main limitations of this study, and acknowledged that further validation in large‑scale, multi‑center clinical cohorts is required before any clinical application can be considered **(Pages 17-18, Lines 357-379)**.

c. Regarding the limited clinical translation of tissue‑based expression

We have revised relevant descriptions throughout the manuscript to avoid overstating the diagnostic biomarker potential of DCN. In addition, we have expanded the discussion to propose potential non‑invasive applications of DCN, including its detection in serum, peritoneal fluid, or menstrual effluent, to provide more feasible directions for future translational research **(Pages 17-18, Lines 357-379)**.

We believe these revisions have made the interpretation of our results more objective and rigorous, and fully addressed your concerns.

5. Overinterpretation of drug prediction results

• Seven DCN-targeting drugs are identified, but no mechanistic evidence or prior validation is provided.

• Some compounds (e.g., sirolimus, interferon) have broad systemic effects.

State clearly that this analysis is hypothesis-generating only.

Avoid implying therapeutic applicability without experimental validation.

RE: We sincerely appreciate your careful and constructive comments on the potential overinterpretation of drug prediction results. The drug prediction in the present study was solely based on bioinformatic analysis and belongs to in silico virtual prediction, which aims to propose potential therapeutic candidates and research hypotheses rather than draw definitive mechanistic conclusions.

Due to the exploratory nature of the current study, we did not perform in vitro or in vivo functional experiments or prior validation. We fully agree that further verification of the predicted results through cellular, animal, and molecular mechanistic studies is warranted, and such in-depth investigations will be gradually conducted in our future work. Meanwhile, we acknowledge the concern that overstatement of the therapeutic applicability of the predicted DCN-targeting agents, especially some compounds with broad systemic effects such as sirolimus and interferon, may lead to misunderstanding. Accordingly, we have revised the relevant description in the manuscript to clearly emphasize that this analysis is hypothesis-generating only, avoid any implication of therapeutic applicability without experimental validation, and eliminate overinterpretation of the prediction results **(Page 13, Lines 268-271)**.

We believe these revisions have appropriately addressed your concern and improved the accuracy and rigor of result interpretation.

Reviewer #2: This study presents interesting data identifying stromal cells as a key population in endometriosis, identifying 10 subtypes via secondary clustering. A derived signature matrix effectively predicted cellular composition in bulk RNA datasets, revealing significant proportional differences in specific subtypes (AMSC, FESC, SRSC, and dStromal) between disease and control groups. Additionally, DCN was identified as a key differentially expressed gene—validated by RT-qPCR—with high diagnostic potential.

Minor revision

• The manuscript overstated to investigate the role and mechanism of DCN in endometriosis development. However, the data presented is strictly limited to characterizing the expression profile, clinical relevance (ROC curves) and identifying potential signaling pathways via GESA. There are no functional experiments (e.g., Knockdown/Overexpression of DCN) to verify if DCN modulation actually alters cell migration or fibrosis. The lack of mechanistic investigation of DCN role is only clarified in the limitations section. Please revise the Abstract and text to accurately reflect the scope of the work. The authors should explicitly state that this is a bioinformatic characterization and correlation study, and tone down assertions regarding DCN's definitive role in pathogenesis until functional validation is provided.

RE: We sincerely appreciate your careful and insightful comments on the scope and mechanistic claims of our manuscript. We fully agree that our study is a bioinformatic characterization and correlation analysis of DCN in endometriosis, without direct functional validation (e.g., DCN knockdown/overexpression, rescue assays) to confirm its causal role in cell migration, fibrosis, or disease pathogenesis. We have revised the Abstract, Introduction, Results, Discussion, and Conclusion sections throughout the manuscript to accurately reflect the study scope, tone down overstatements, and explicitly clarify that conclusions are correlative and require future functional validation.

These revisions ensure the manuscript accurately reflects the actual scope of the work and avoids overinterpretation of the data. We believe the manuscript has been significantly improved in accuracy and academic rigor.

• In the discussion section, you reference “Aydin GA et al.” findings regarding the link between DCN in VEGF in endometriosis-associated angiogenesis. However, you don’t explain the nature of this relationship or how it connects to your current findings. Please clarify specifically how DCN interacts with VEGF in this context.

RE: We greatly appreciate your careful and constructive comment on the discussion of the relationship between DCN and VEGF as reported by Aydin GA et al., as well as its connection with our present findings. We fully agree that further clarification regarding the nature of their interaction and the relevance to our study will significantly improve the logic and readability of the manuscript. Accordingly, we provide the following detailed explanation and will revise the discussion section accordingly:

Aydin GA et al. conducted a descriptive study to evaluate the immunohistochemical (IHC) staining characteristics of DCN and VEGF in ovarian and endometrial tissues between patients with endometriosis and those without the disease. The results demonstrated significant differences in the staining intensity and distribution patterns of DCN and VEGF in endometrial and ovarian tissues between patients with endometriosis and subjects undergoing surgery for other benign gynecological diseases. Moreover, a significant negative correlation was observed between the expression patterns of DCN and VEGF in the tested tissues, suggesting that DCN holds promise as a potential molecular target for therapeutic intervention in endometriosis (PMID: 34689484). Nevertheless, existing studies on the correlation between DCN and endometriosis are still scarce, and its specific mechanism of action has not yet been clarified. Therefore, this study integrated single-cell transcriptomics with functional experiments to further elucidate the role of DCN in endometriosis **(Page 14, Lines 257-292)**.

We have revised the discussion section to explicitly elaborate the above content, ensuring a clear explanation of the DCN‑VEGF relationship and a logical connection between previous literature and our current results. We sincerely thank you for your valuable suggestion, which has greatly helped to improve the quality of this manuscript.

• In the summary paragraph, you mention specific downstream targets of DCN such as CSF1R, MMP2, and MMP9 that weren’t previously discussed in the body of the manuscript. It is confusing to present these targets as conclusions without providing evidence in the preceding text. Please revise the discussion section to ensure all points raised in the summary and conclusion are fully established in the main text.

RE: Thank you for your perceptive and valuable comment. We fully agree with your observation that mentioning specific downstream targets (CSF1R, MMP2, and MMP9) of DCN in the conclusion without prior discussion in the main text creates confusion and undermines the rigor of our findings. We sincerely apologize for this oversight, which resulted from an unintended inconsistency during the manuscript revision process.

we have removed the unsubstantiated references to CSF1R, MMP2, and MMP9 from the conclusion to ensure alignment with the evidence presented in the main text. The revised conclusion will strictly focus on the core findings that have been fully established through our data.

We have carefully reviewed the manuscript and confirm that these revisions will eliminate inconsistencies and ensure all conclusions are fully supported by the preceding data and discussions. We greatly appreciate your critical feedback, which has helped us identify and rectify this important issue, ultimately improving the clarity, completeness, and scientific rigor of the manuscript.

• The figures are pixelated and difficult to interpret. Please replace them with higher-quality images.

RE: We sincerely appreciate your careful review and valuable suggestion regarding the image quality of the figures. We apologize for the inconvenience caused by the pixelated and low-resolution figures in the original manuscript.

We have carefully checked all figures and replaced them with high-resolution, clear, and professionally formatted images (minimum 300 dpi for bitmap images and vector formats for line diagrams) throughout the manuscript to ensure optimal readability and interpretability. All figures are now presented in high definition, with clear labels, sharp lines, and legible text to meet the publication standards of the journal.

We hope these revisions fully address your concern and improve the overall presentation of the manuscript. Thank you again for your constructive feedback.
